# Salinity as a Determinant Structuring Microbial Communities in Coastal Lakes

**DOI:** 10.3390/ijerph19084592

**Published:** 2022-04-11

**Authors:** Sylwia Lew, Katarzyna Glińska-Lewczuk, Paweł Burandt, Klaudia Kulesza, Szymon Kobus, Krystian Obolewski

**Affiliations:** 1 Department of Microbiology and Mycology, University of Warmia and Mazury in Olsztyn, Oczapowskiego Str. 1a, 10-719 Olsztyn, Poland; klaudia.kulesza@uwm.edu.pl; 2 Department of Water Management and Climatology, University of Warmia and Mazury in Olsztyn, Łódzki Sq. 2, 10-719 Olsztyn, Poland; kaga@uwm.edu.pl (K.G.-L.); pawel.burandt@uwm.edu.pl (P.B.); szymon.kobus@uwm.edu.pl (S.K.); 3 Department of Hydrobiology, Kazimierz Wielki University in Bydgoszcz, Powstańców Wielkopolskich Str. 10, 85-090 Bydgoszcz, Poland; obolewsk@ukw.edu.pl

**Keywords:** microbial communities, salinity, coastal lakes

## Abstract

The response of bacterioplankton structure to salinity level in coastal lakes (*n* = 9) along the southern Baltic Sea coastline was studied. In terms of mean salinity levels (0.2–5.2 PSU), the lakes represented freshwater, transitional, and brackish types. Results showed that salinity determines the spatial and seasonal distribution patterns of microorganisms in costal lakes. Increased salinity contributed to a significant decline in total bacterial numbers (TBN). The TBN was lowest in brackish lakes in autumn (4 × 10^6^ cells/mL) and highest in freshwater lakes in summer (7.11 × 10^6^ cells/mL). The groups of *Proteobacteria* are appropriate bioindicators in any classifications of coastal ecosystems, particularly at low-haline stress. *Alpha*- and *Gamma*- subclasses of *Proteobacteria* are identifiers for brackish habitats, while *Betaproteobacteria*, due to their intolerance to haline stress, prefer freshwater habitats. Counts of euryhaline *Actinobacteria*, the dominant group of bacterioplankton (31.8%), decreased significantly with increased salinity. *Actinobacteria* and *Deltaproteobacteria* were identifiers of transitional lakes. *Cytophaga-Flavobacteria* showed affinity with freshwater ecosystems, but this relation was not statistically significant (*p* > 0.05). The bacteria groups correlated with other physico-chemical parameters of water, such as oxygenation (*Actinobacteria*) or organic carbon (*Betaproteobacteria*, *Deltaproteobacteria*). The impact of hydrological connectivity and salt-water interference on the microbiota structure and biogeochemistry of coastal waters should be considered in the assessment of the ecological status of coastal lakes.

## 1. Introduction

The abundance and diversity of microorganisms inhabiting water systems both regulate and indicate the health or condition of water ecosystems [1,2]. The spatial patterns of microorganism structures typically have a strong heterogeneous distribution in coastal habitats as a consequence of the natural variation in physico-chemical properties of water, temperature fluctuations, and tide actions [3,4,5]. Understanding the response of microorganisms to environmental factors is of great importance for comprehending the acclimatisation and evolution of microorganisms in natural environments [6]. Among the abiotic factors, salinity is known as a conservative tracer, and as having the most influence on aquatic ecology. It determines the composition of microbial communities, more so even than the impacts of temperature and pH [7,8,9]. Salinity affects the physiological properties of cells, strengthens their osmotic potential, reduces the activity and biomass of microorganisms and, thus, significantly affects the structure of communities [10].

Coastal lakes are specific habitats that, despite their close proximity, differ by hydrological connectivity with the sea [10] ecosystems are judged as important sites of biodiversity, biological productivity, and ecosystem services such as the removal of sediment, nutrients, and contaminants from inflowing rivers [11]. Their habitat values are considered by the EU Natura 2000 system in Annex I and II of Habitat Directive [12] as a priority habitat protection with the code 1150-2, which denotes coastal lakes. The functioning of coastal ecosystems is, however, dependent on many threats from the land (pollution, tourism) and the sea (salt-water intrusion related to the intensity of sea storms and associated backwater). They are deemed, due to their location along the coastline, to attest directly to climate change: they are recipients of saline water intrusions due to a rise in the seawater level. The predicted effects of global climate change are likely to intensify saline intrusions into such ecosystems [13] as the global mean sea level is projected to rise by 0.09 to 0.88 m by 2100 [14], thereby salinising transitional and many freshwater coastal aquatic ecosystems. Due to increasingly frequent intrusions of seawater, biotic structure and trophic chain in the biotopes of many coastal lakes and wetlands are likely to alter, also contributing to significant changes in ecosystem services [12]. The development and functioning of shallow lagoons, estuaries, mangroves, wetlands, and lakes located in the coastal zone of seas and oceans is conditioned by hydrological conditions and the degree of intrusion of seawater [10]. Thus, one of the criteria for classifying these environments is the degree of salinity, on which the Venice System [15] is based. The division of coastal ecosystems results from the intensity and duration of seawater intrusion. Thus, lakes, lagoons, or estuaries may be classified to various types of habitats, even at low salinities: freshwater (also known as limnetic < 0.5 PSU), where the ecosystem is isolated from seawater intrusions; transitional (known also as oligohaline, with 0.5–5.0 PSU), which are under the influence of marine water at high backward flows, and brackish (also known as mesohaline), with variable salinities of 5–18 PSU.

Coastal water bodies located in the vicinity of the Baltic Sea, one of the world’s low-salinity seas [16,17], are habitats characterised by salinity <5 PSU, and thereby they are well suited as model objects for studying indigenous organisms, including microbiota, exposed to low-haline stress [18,19,20]. It is worth noting that the Baltic Sea is a highly polluted and eutrophicated ecosystem, and hence a productive and complex one [18,21]. In brackish waters, the community of microorganisms is much poorer than in fresh or marine waters. Even low-haline stress may influence the diversity of bacterioplankton through the presence of bacteria with varying degrees of ecological specialisation [22]. To survive in unfavourable or changing habitat conditions, bacteria use two main ecological strategies, each based on the presence of one of two functional groups of microorganisms in the microbiome: generalist and specialist habitats. A specialist habitat is generally more sensitive to changes in the environment due to its strong adaptation to the local environment, while a generalist habitat is more resilient to changes [23,24]. Environmental changes imply the rebuilding of the microbial community, which results in the emergence of a large number of specialist habitats, and their participation seems to be mainly influenced by limiting factors. On the other hand, environmental variability caused by periodic changes in salinity, even small ones, in transitional lakes favours a generalist habitat, which has broad environmental tolerance and responds to stochastic factors [24,25]. Any changes, including those related to abundance (mainly its reduction), as well as changes in diversity, are an indicator of habitat disturbance.

Research on the impact of salinity on the communities of coastal ecosystems has not yet explained its influence on the full structure of bacterioplankton [24]. This may result from stress caused by both anthropogenic pollution and the pressure of environmental parameters [25,26]. Despite the growing interest in the influence of abiotic factors on the structure of microbial communities in coastal ecosystems [6,17,27,28,29] biotic changes in the low-salinity ecosystems still require more attention of researchers [30,31,32]. Observations of changes in the structure of bacterioplankton within coastal lakes would also allow the creation of a model for the early detection of changes in freshwater-to-brackish ecotone zones within a global range. Due to the key role of microorganisms in a variety of ecological processes, from primary production to organic carbon transformation, the structure of bacterial assemblages in response to salinity stress will provide further knowledge on these important ecosystems.

The aim of the study was to assess the role of salinity gradients in coastal lakes for the structure of bacterioplankton communities, at the level of basic microbial groups. For this purpose, we verified the following hypotheses: (i) water salinity level is a basic determinant structuring microbiological communities of coastal lakes; (ii) bacteria groups show various ecological tolerance not only to salinity changes, but also to other abiotic indicators determining the condition of coastal ecosystems.

## 2. Materials and Methods

### 2.1. Study Area

The study was conducted on nine coastal lakes located along the southern Baltic Sea coast. The lakes are shallow and have a poorly developed shoreline. They are polymictic and eutrophic, but they differ in morphometry, hydrology and the physico-chemical properties of their water (Table 1) [13,14]. Based on the differences in surface connectivity with the sea, and thus in exchange of seawater and salinity level [10], they are classified into three salinity groups, according to the Venice system [15]. They represent the following types of habitats: (i) freshwater—with no connection to the sea, and salinity at the limnetic level (Dołgie Wielkie, Sarbsko, Wicko Przymorskie); (ii) transitional—periodically fed with seawater of mixed salinity and limnetic/oligohaline character (Liwia Łuża, Gardno, Kopań); (iii) brackish—continuously fed with seawater with an average salinity level defined as mesohaline (Łebsko, Ptasi Raj, Resko Przymorskie).

### 2.2. Sampling

**Figure 1:**
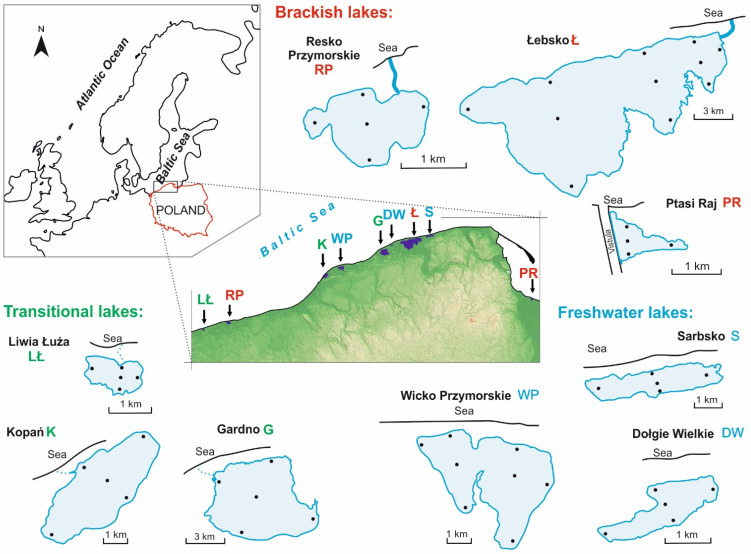
Location of studied coastal lakes along the Southern Baltic Sea. Black dots indicate sampling sites.

For each year of the project, samples were taken three times a year, during growing seasons, at three-month intervals: in April (spring), July (summer), October (autumn). The samples were collected from the subsurface layer from four to seven stations per each lake depending on water table area and morphological diversity (Figure 1). In total, 282 microbiological samples were analysed. Simultaneously with the collection of water samples for microbiological analyses, in situ measurements of the physico-chemical properties of water were performed, and water samples were collected for physico-chemical laboratory analyses.

### 2.3. Physico-Chemical Analyses of Water

Physical and chemical parameters of water were measured at each study site using a multi-parameter YSI 6600 probe (YSI Inc., Yellow Springs, OH, USA) and AP-7000 (Salinas, London, UK) at each sampling: water temperature (T, °C), pH, dissolved oxygen (DO, %), Chl-*a* (µg/L), salinity (PSU), and conductivity (EC, µS/cm). Water samples for the laboratory analyses were collected from all study sites according to the methodology described by Obolewski et al. [10]. Water samples from all study sites were analysed in laboratory for N-NO_2_^−^, N-NO_3_^−^, N-NH_4_^+^, TP (total phosphorus), P-PO_4_^3−^, total organic carbon (TOC), and dissolved organic carbon (DOC) with the methodology described by Obolewski et al. [10]. Total inorganic nitrogen (TIN) is a product of summarised concentrations of N-NO_2_^−^, N-NO_3_^−^, and N-NH_4_^+^.

### 2.4. Microbial Community Analyses

Total bacterial number (TBN) was determined by direct epifluorescent filter technique (DEFT) [30]. Lake samples were taken in triplicate to determine the variability of DAPI counts. Samples were fixed with neutralised formaldehyde (pH 7.4) at a final concentration of 4% and stored at 4 °C to perform the analysis. Staining was performed within one week of sampling. Subsamples were stained with 4,6-diamidino-2-phenylindole (DAPI Sigma-Aldrich, St. Louis, MO, USA), final concentration (1 µg/mL) for 15 min in the dark and gently filtered through 0.2 µm black Nuclepore filters (type GTTP, Millipore). The bacteria were counted using a BX41 (Olympus, Japan) epifluorescence microscope. More than 1000 bacterial cells were counted in 20 fields of vision.

The community composition of bacterioplankton was analysed by fluorescent in situ hybridisation DOPE-FISH (double labelling of oligonucleotide probes) [33,34,35,36]. Samples were fixed in freshly prepared buffered paraformaldehyde (pH 7.4) to a final concentration of 4% (vol/vol) and stored for several hours at 4 °C. The samples were filtered through white polycarbonate filters (type GTTP; Millipore), rinsed twice with sterile water, dried at room temperature, and stored at −20 °C.

The bacterial community was analysed using an EUB338I-III oligonucleotide probe, targeting total bacteria [30]. Within the bacteria, the main taxonomic groups were determined using: ALF968, BET42a, GAM42a, and DELTA 495abc probes for *Alpha-*, *Beta-*, *Gamma-*, and *Deltaproteobacteria*, respectively [36,37]; a CF319a probe for *Cytophaga-Flavobacteria* [31]; and a HGC69a probe for *Actinobacteria* [38]. The characteristic oligonucleotide probes used in this study are presented in Table 2. Autofluorescence and non-specifically stained cells were determined with a NON338 negative control probe [30]. All oligonucleotide probes were double labelled with Cy3 dye [35]. Bacterial cells on the filter sections were observed with an epifluorescence microscope equipped with filter sets for DAPI and CY3 [39]. The fractions of DOPE-FISH-stained bacteria in at least 1000 DAPI-stained cells per sample were quantified in triplicate. Probe-specific cell counts are presented as the percentage of cells visualised by DAPI.

### 2.5. Statistical Analyses

Data were analysed to: (a) characterise and compare seasonal composition and density of microbiological communities in nine coastal lakes, representing three habitat types distinguished by salinity levels; (b) relate the natural environmental factors with the microbiological communities’ structure and distribution in the nine coastal lakes; and (c) identify microbiological indicators for each of the three salinity types of coastal lakes. The responses of the microbiological communities to the environmental variables were analysed using multivariate statistical analyses. Prior to the statistical analyses, the normal distribution of the microbiological data and environmental variables were tested with the Kolmogorov–Smirnov test. The datasets were log_10_(x + 1) transformed to stabilise the variance. A one-way analysis of variance (ANOVA) with Tukey’s multiple comparison test (*p* < 0.05) as a post hoc procedure was used to test differences between functional groups of bacteria between seasons and lake types. We also ran a two-way ANOVA to test differences in the bacteria groups and environmental variables in seasons. The correlation analysis, mean abundances, and standard deviations (±SD) were calculated using Dell™ Statistica™ 13.1. A principal coordinates analysis (PCoA) using a normalised Euclidean distance was applied to justify spatial differences in physico-chemical parameters of the three lake-habitat types (freshwater, transitional, brackish), and Bray–Curtis distance for microbiological data [40]. Dissimilarity between lakes and lake types was determined using the similarity percentage analysis procedure (SIMPER) based on the Bray–Curtis dissimilarity metrics [40,41]. PCoA and SIMPER analyses were performed in PAST v3.05 [42]. To confirm the reliability of using salinity as a criterion of habitat classification of the studied coastal lakes, we used two-way cluster analyses (TWCA) in PC-ORD 6.08 [43] with Sorensen’s (Bray–Curtis) distance measure and flexible-β set to −0.25 as the linking algorithm. The data were relativised by maximum. The clusters were combined into a single heat map to enable associations between variables to be visualised. Heat map colours and size indicate minimum (white) to maximum (blue) contribution of each bacterioplankton group in the community.

The specific microbiological groups for a given lake type were determined by indicator species analysis (ISA), (*p* < 0.01). ISA is a simple and useful method to identify indicator species and/or species assemblages showing the specificity of habitats [43]. Indicator values (IndVal) were calculated based on abundance of microbial community group in relation to coastal lake type and expressed as the product of the specificity and fidelity.

To visualise interrelationships between the groups of microorganisms (biological features) and chemical parameters of brackish lakes, we used a chord diagram. This was generated on the basis of a matrix of normalised sums of biological features values in the entire lake type and expressed in %. The R environment was used to create the diagram, and the RStudio program was used with the library chorddiag by Matt Flor.

## 3. Results

### 3.1. Physico-Chemical Parameters of Water

In the studied lakes, we noted conductivity and salinity within the ranges of 86–13,228 µS/cm and 0.09–10.26 PSU, respectively (Table 3). Such a range of data was appropriate to classify them according to the Venice criterion into: freshwater, transitional, and brackish lakes with mean salinities of 0.24 (±0.15 SD), 1.17 (±0.86 SD), and 5.22 (±2.83 SD) PSU, respectively. Salinity was more than 21-fold higher in habitats brackish than in freshwater. Lakes that are connected to seawater, i.e., brackish and transitional ones, showed significantly higher concentrations of mineral forms of nitrogen compared to the waters of freshwater lakes. The transitional lakes are characterised by the highest values of pH and DO (*p* < 0.05). Brackish lakes, on the other hand, have significantly lower, almost by half, mean concentrations of TOC and DOC compared to other types of lakes. The studied lakes showed relatively uniform concentrations of phosphorus in water. The dataset had a range of 0.10–0.14 mg/L for phosphates and 0.34–0.40 mg/L for TP.

Clear seasonal patterns (Table S1—Supplementary Material) were observed for all water variables except TP (two-way ANOVA: season * type, *p* < 0.05). At most of the study sites, salinity reached the highest mean values in autumn, amounting to 0.21 PSU for freshwater, 1.02 PSU for transitional, and 4.06 PSU for brackish lakes. Salinity was significantly higher in autumn than in spring (*p* < 0.001) regardless of lake type. This is attributed to the strong winds blowing from the sea and the backwater that pushes seawater inland causing the resuspension of bottom deposits. This also explains the high values of water aeration, chlorophyll-a, N-NO_3_^−^, and TIN, which also increase significantly in autumn.

**Figure 2:**
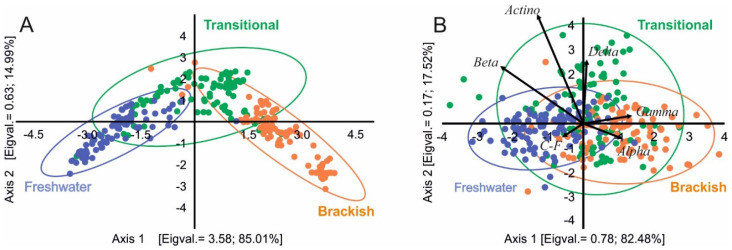
Principal coordinates analysis (PCoA) for physico-chemical parameters of water (**A**) and microbiological data (**B**).

The PCoA confirmed remarkable differences between the coastal lakes predetermined as three lake habitats: freshwater, transitional, and brackish water. The analysis also revealed that the three types distinguished on the physico-chemical properties of water (Figure 2A) are consistent with the habitat preferences of microbiological groups (Figure 2B).

### 3.2. Bacterioplankton Characteristics in Coastal Lakes

The mean value of TBN for the studied coastal lakes amounted to 5.79 × 10^6^ (±3.19 SD). The three lake types differed statistically in terms of total bacteria number (TBN), (one-way ANOVA, post hoc: Tukey’s HSD test; *p* < 0.05). The smallest number of microorganisms characterised brackish lakes (4.78 × 10^6^ (±2.56 SD)) and did not differ statistically from transitional lakes (5.48 × 10^6^ (±2.90 SD)). The group of ecosystems isolated from the sea (freshwater) showed significantly higher TBN (6.93 × 10^6^ (±3.58 SD)) than other types of lakes (Table 4). The dominant group of microorganisms in all types of lakes was that of *Actinobacteria*, which was recorded in its lowest numbers in lakes with permanent contact with seawater, i.e., in brackish lakes (Table 4). *Actinobacteria* accounted for 26.8–31.8% of DAPI-stained cells (Table 4, Figure 2), which equalled 2.6–6.6 × 10^5^ cells/mL. A similar pattern was noted for the second most numerous group of microorganisms, *Betaproteobacteria*, but they were most frequently recorded in freshwater lakes. The group’s share in TBN amounted to 17.3% (±4.7). The mean value for the transitional lakes did not differ statistically significantly and amounted to 16.1% (±6.3). In brackish lakes, *Cytophaga-Flavobacteria* appeared more often in the community. Their number in TBN did not differ statistically between the studied groups of lakes and ranged from 14.06% in transitional ones to 15.2% in freshwater ones, which corresponded to 0.8 × 10^6^ and 1.1 × 10^6^ cells/mL, respectively. *Alpha-* and *Gammaproteobacteria* did not belong to the abundantly represented groups of the studied bacterioplankton. Nevertheless, they were more often recorded in brackish lakes, where their counts were significantly higher when compared to the other two types of lakes (Table 4). *Alphaproteobacteria* accounted for 4.4–6.7% of DAPI-stained cells (Table 4, Figure 2), which equalled 0.30–0.32 × 10^5^ cells/mL, whereas for *Gammaproteobacteria* it was 9.5–12.2% of DAPI-stained cells, which constitutes 0.66 and 0.59 × 10^5^ cells/mL, respectively.

**Figure 3:**
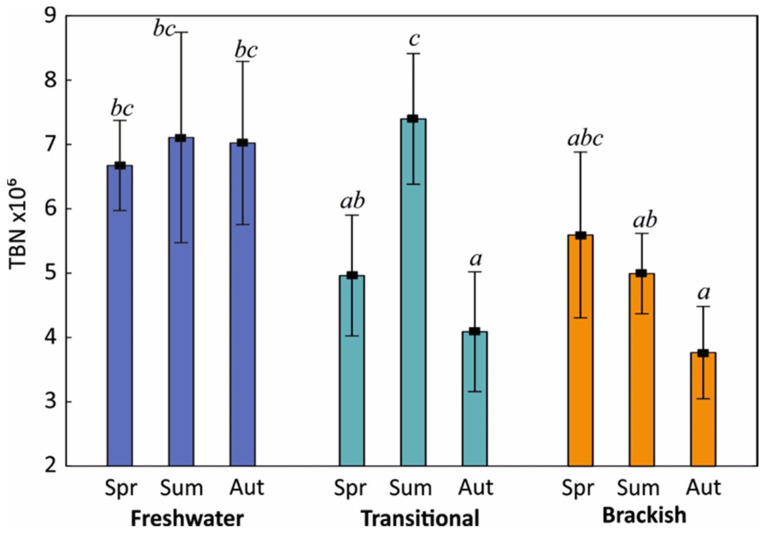
Changes in TBN (×10^6^ cells/mL) in relation to lake types and seasons. Different superscript letters indicate statistically significant differences between the groups of lakes (two-way ANOVA, Tukey’s HSD test (*p* < 0.05) as a post hoc procedure).

The quantitative structure of bacteria differed significantly between seasons within lake types (Figure 3). In lakes characterised by the largest variability of water exchange with the sea (transitional), changes in bacteria counts were linked with the presence of hydrological phases (limnic vs. oligohaline). Thus, the greatest variation in the number of microorganisms during the year was found in transitional lakes. In the summer, when the intrusion of seawater was lowest (limnic phase), the values of TBN in these lakes were the highest (averaging 7.40 × 10^6^ cells/mL) and they did not differ significantly from the value of this parameter for freshwater lakes (7.11 × 10^6^ cells/mL), (HSD test, *p* < 0.05). The connection with the sea being permanent or periodic (oligohaline phase) did not favour bacteria activity, particularly in autumn, when the TBN values were lowest, at approx. 4 × 10^6^ cells/mL. A similar trend was observed in spring, but these differences were not statistically significant (*p* < 0.05).

The results of similarity percentage analysis (SIMPER) (Table 5 and Table S2) identified the main groups of bacteria that typified a given lake according to the salinity gradient. SIMPER showed that even relatively small changes in salinity stress seem capable of altering the bacterial structure. In terms of average dissimilarity, the Proteobacteria groups formed the following decreasing order: *Actino* > *Beta* > *C-F* > *Delta* > *Gamma* > *Alpha*. The highest contribution (27.7%) to the similarity between lake types was for *Actinobacteria*, which represents a euryhaline group of organisms. The contribution of *Alphaproteobacteria* was the lowest (7.2%), particularly in freshwater lakes (4.4%).

### 3.3. Environmental Determinants of Bacterial Communities Structure

**Figure 4:**
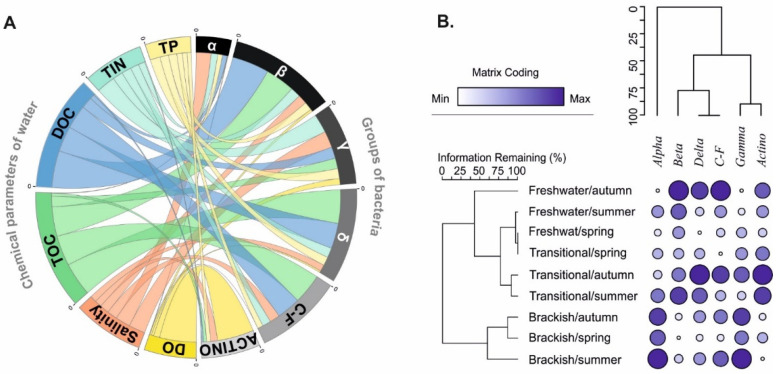
(**A**) Chord diagram of relations between chemical parameters of waters and groups of bacteria (α—Alphaproteobacteria; β—Betaproteobacteria, γ—Gammaproteobacteria, δ—Deltaproteobacteria, C-F—Cytophaga-Flavobacteria, Actino-Actinobacteria) in brackish lakes. (**B**) Two-way cluster analysis (TWCA) based on the relative value of bacteria groups in samples from three types of coastal lakes in seasons. Cold map colours indicate minimum (white) to maximum (blue) contribution of bacteria groups.

The observed diversity of bacterial communities required the search for mutual relations between microorganisms and selected physicochemical parameters for each group of lakes. Among the physico-chemical parameters that quantitatively regulate the community structure and abundance of microorganisms in coastal lakes, salinity and carbon content (TOC and DOC) had the strongest influence, as visualised in the chord diagram (Figure 4A).

Salinity was the parameter that contributed most to the decrease in TBN in coastal lakes (r = −0.26, N = 282, *p* < 0.001)). It negatively impacted *Beta* (r = −0.41), *Actino* (r = −0.27, *Delta* (r = −0.21) and *C-F* (r = −0.16) in particular, whereas *Gamma-* and *Alphaproteobacteria* positively corresponded to the salinity increase (r = 0.36 and r = 0.29, respectively). The highest inverse correlation between salinity and *Alphaproteobacteria* counts (% of TBN) was noted in freshwater lakes (r = −0.36; *p* < 0.001), and *Betaproteobacteria* (r = −0.34; *p* < 0.001). Periodic intrusion of seawater had a positive influence on the number of microorganisms in bacterioplankton in transitional lakes (r = 0.21; *p* < 0.05). The only groups of bacterioplankton positively correlated with the salinity were C-F in transitional lakes (r = 0.23, *p* < 0.05) and *Gammaproteobacteria* in brackish lakes (r = 0.45; *p* < 0.001).

Organic carbon in water determined the presence of *Betaproteobacteria* (Figure 4A) regardless of lake-habitat type, with correlation indices of r > 0.90 for DOC and r > 0.75 for TOC. Similarly, positive response to DOC and TOC were shown by *Deltaproteobacteria*, though at lower significance levels. In the latter, it was observed that the increase in water temperature stimulated the multiplication of microorganisms and an increase in their number (TBN) in the community (r = 0.38, *p* < 0.05). The occurrence of *Actinobacteria* was largely correlated with the saturation of water DO (r = 0.89). A similarly strong dependence (r = 0.87) was noted in individual types of lakes, regardless of the degree of connection to the sea. *Alphaproteobacteria*, which were most abundant in lakes under the direct influence of seawater, favoured increased water temperatures (r = 0.57), but avoid higher concentrations of TOC, DOC, and TP. In all water bodies, *Gammaproteobacteria* was recorded at a higher concentration of TIN.

Results of two-way cluster analysis (TWCA) based on seasonal changes in water salinity in the three types of lake indicated three clusters of bacteria groups (Figure 4B). Among them, an individual cluster of *Alphaproteobacteria* corresponds with a cluster formed by brackish lakes. *Betaproteobacteria*, *Deltaproteobacteria*, and *Cytophaga-Flavobacteria* formed a cluster corresponding with a cluster formed by freshwater and transitional lakes in summer and autumn. *Gammaproteobacteria* and *Actinobacteria* formed a cluster specific for lakes with periodical and permanent seawater supply (transitional and brackish) in summer and autumn, mainly. From the TWCA, one may conclude that salinity-related and seasonal differences caused phylogenetically clustered shifts in bacterial community composition.

## 4. Discussion

In Baltic coastal lakes, bacterioplankton, as the lowest group in the trophic chain, is sensitive to haline stress (Table 2). Similarly to other saline-sensitive bioindicators such as benthic invertebrates [44,45,46] or phytoplankton [10], microbial communities respond to changes in salinity even at relatively low values. Our results showed that a relatively narrow salinity gradient (0.2–5.2 PSU) structured bacterial communities living under limnetic, oligohaline, and mesohaline conditions of coastal lakes. Our comparisons between the structure of prokaryotic communities along the salinity gradient are generally consistent with salinity-driven global patterns of microorganism structures [6,19]. We have found the prevalence of *Betaproteobacteria* in the freshwater type of lakes to the dominance of *Gamma*- and *Alphaproteobacteria* in the brackish type. *Actinobacteria* seem to be more euryhaline organisms, though they follow a similar trend to *Betaproteobacteria*, which decreases with increasing salinity [47,48], whereas the abundance of the *Cytophaga-Flavobacteria* [49] do not show a clear relationship with salinity [50,51]. Our study is one of only few reports dealing with *Deltaproteobacteria* in coastal ecosystems. Our results demonstrated a statistically significant negative correlation of *Deltaproteobacteria* with salinity [52], (Table S1).

To sum up the effects of exposure of bacteria groups to haline stress, in the Indicator Species Analysis (Table 6), we found that the studied groups of bacteria are appropriate indicators of the salinity of the environment (*p* = 0.014): *Alpha-* and *Gammaproteobacteria* as bacteria group identifiers for brackish lakes (such as Resko, Łebsko or Ptasi Raj), *Actinobacteria* and *Deltaproteobacteria* for transitional lakes (Gardno, Kopań, Liwa Łuża), and *Betaproteobacteria* for freshwater coastal lakes (such as Dołgie Wielkie, Sarbsko, Wicko). *Cytophaga-Flavobacteria* show affinity with freshwater ecosystems, but this relation was not statistically significant (ISA, *p* = 0.08).

*AlAlphapAlphaproteobacteria* and *Gammaproteobacteria* show a preference for environments with brackish waters (>5 PSU), which comprise a significantly higher % of TBN (6.7 and 12.2, respectively). *Alpha-* are known to prefer warm, oxygenated waters with high exposure to sunlight and low availability of organic nutrients [53], whereas *Gammaproteobacteria* prefer water rich in nutrients and are found in large numbers in the deeper layers of lakes. Our study showed that *Gamma-* is the second least numerous group of microorganisms (after *Alpha-*), positively correlated with N-NO_2_^−^ and N-NO_3_^−^, thus indicating that these bacteria are copiotrophs, i.e., organisms able to quickly utilise nutrients [54].

*Actinobacteria* are a main actor among bacterioplankton communities, constituting of from 30% to 70% TBN and thus have a significant role in nutrient and energy cycling in these ecosystems [49,55,56]. We noted the largest share of this group in transitional (31.8%) and freshwater (30.1%) lakes. The dominance of *Actinobacteria* in all the studied reservoirs may suggest the freshwater nature of the studied lakes. This limited contact with saline water seems to somehow stimulate the development of *Actinobacteria*, because the salinity >5 PSU significantly (*p* < 0.001) reduces their abundance in brackish lakes, as reported by, e.g., Holmfeldt et al. [56]. The presence of *Actinobacteria* in the community in transitional lakes favours water aeration (DO) and higher water temperatures in summer. *Actinobacteria* abundant in this type of coastal lakes are, on the one hand, evidence that a significant part of these bacteria constitutes the freshwater-limnetic HGC-IA cluster; on the other hand, it proves the high environmental adaptability of these organisms. Even low salinity levels can cause oxidation stress that can alter bacterial composition, with consequences that will resonate throughout the ecology of coastal ecosystems [57].

*Deltaproteobacteria* have the ability to ferment or reduce iron or sulphates and are usually found in deeper water layers. In our opinion, due to the lack of a significant correlation between this class of bacteria and the physico-chemical parameters of water in the lakes studied, the presence of these bacteria in bacterioplankton exceeding on average 10% of Procaryota is the result of the polymictic nature of the studied lakes [30].

The *Beta*-subclass of *Proteobacteria* dominated in the bacterioplankton of freshwater type of coastal lakes (comprising on average 17.3% of TBN), which is not surprising because of its dominance among bacterioplankton in freshwater ecosystems [48]. The abundance of *Betaproteobacteria* in coastal lakes was reduced by salinity, but positively influenced by the content of dissolved organic carbon [16,30,48], and oxygen content. Aeration is the most important parameter explaining the quantitative composition of the communities of microorganisms in all the studied lakes. However, the seawater intrusion can be a significant threat for this group of bacteria, as also shown by Zhang et al. [6]. This can be clearly seen in the example of transitional lakes, where the decline in *Betaproteobacteria* as well as *Cytophaga-Flavobacteria* may be the result of physiological stress caused by the mixing of waters of different salinities [58].

Nevertheless, the lack of statistically significant differences between *Cytophaga-Flavobacteria* abundance in the studied lake types (ISA, *p* > 0.05) suggests that the salinity is not the main factor determining changes in this eurytypic community [48]. The ability of these microorganisms to mineralise complex macromolecules, particularly common in the autumn [30], is confirmed by the seasonal dynamics models and high positive correlation values with TOC and DOC in our analyses. It is worth noting that *Cytophaga-Flavobacteria* are reported to also be regulated by *Cyanobacteria* blooms [59]. The supply of carbon and organic nitrogen through the inflow of allochthonous organic matter created favourable conditions for the development of bacteria in marine coastal zone waterbodies. Quantitative changes in the communities of microorganisms are related to the seasonal supply of organic carbon [60]. Therefore, seasonal changes in the structure of the prokaryotic community in the coastal lakes may be the result of the availability of organic matter, the concentration of which is regulated not only by hydrological connectivity with the sea but also input of allochthonous matter from lake catchment [31]. Based on the short generation times of many bacteria, together with their rapid evolution and remarkable trophic versatility, environmental boundaries can be crossed more frequently than is the case for plants or animals [53].

**Figure 5:**
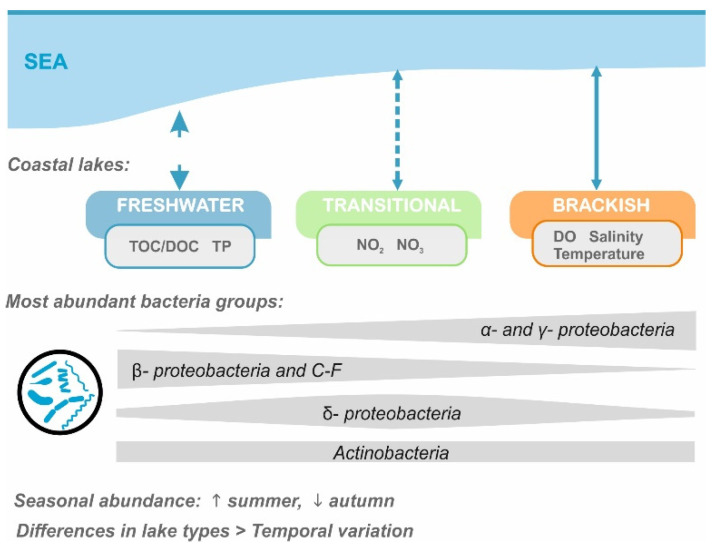
Summary diagram of the response of microbial community structure to the salinity stress and physico-chemical parameters of water in coastal lakes.

Since hydrological connectivity with the sea changes the abiotic parameters of coastal ecosystems [61,62], we have found salinity to be a direct determinant of changes in main groups of prokariota in the coastal lakes along the southern Baltic Sea coastline. Nevertheless, the observed bacterial community composition patterns also result from factors that co-correlate with salinity (TOC, DOC, and DO). The response of microbiological community structure to the salinity stress and physico-chemical parameters of water in coastal lakes is shown in Figure 5.

Our study is the first to develop bacteria assemblage thresholds (reaction) for salinity in the southern Baltic coastal lakes. The spatial and temporal survey of microorganisms along a salinity gradient throughout the coastal lakes can be used to update and harmonise coastal ecosystem protection guidelines by region for more accurate assessments and forecasts of seawater intrusion and related changes in water-quality parameters. Based on the response of the bacteria to increased salinity that we recorded, we inferred that it implies that global climate change scenarios may bring about similar impacts on the bacterioplankton community in the future. These changes may further disturb the ecological functioning of these valuable and vulnerable ecosystems [46].

This study also allowed for the identification of a more complete structure of bacterial assemblages upon which the proper functioning of coastal lakes is founded with regard to the circulation of matter and energy in these ecosystems and the maintenance of their homeostasis.

## 5. Conclusions

The analysis of the bacterial community compositions in relation with the degree of hydrological connectivity of coastal lakes with the Baltic Sea, and thus different concentrations of selected water parameters, showed that even slight differences in salinity model the composition of the communities of microorganisms that constitute bacterioplankton. The presented patterns of community composition in various groups of lakes are a quick, direct response to environmental changes (mainly salinity) and may be indicators that enable modelling of even long-term climate changes. *Alpha-* and *Gammaproteobacteria* prefer waters with higher salinity, *Actinobacteria* and *Betaproteobacteria* prefer freshwater lakes, and, to a lesser extent, very high adjustability of *Cytophaga-Flavobacterium* outlines a structure, exceptions to which may become an alarming factor for the effects of disturbances in the ecosystem. Short-term re-composition of microorganism communities is a quick answer to the question of the direction of changes in the environment of coastal lakes due to climate change. The study, as part of the biomonitoring of coastal lakes, recognised a key area of interest for researchers studying the impact of hydrological connectivity and salt-water interference on the ecology and biogeochemistry of coastal waters.

## Figures and Tables

**Table 1:** Morphometric characteristics and classification of the studied coastal lakes.

Lake Name	Geographic Coordinates	Area (ha)	Mean Depth (m)	Type of Lake According to Venetian Classification	Habitat
Liwia Łuża	54°05′ N, 15°06′ E	172	1.0	Limnetic/oligohaline	Transitional
Resko Przymorskie	54°09′ N, 15°21′ E	554	1.6	Mesohaline	Brackish
Wicko Przymorskie	54°33′ N, 16°38′ E	977	2.4	Limnetic	Freshwater
Kopań	54°29′ N, 16°27′ E	753	1.5	Limnetic/Oligohaline	Transitional
Gardno	54°39′ N, 17°07′ E	2261	1.4	Oligohaline /Limnetic	Transitional
Łebsko	54°43′ N, 17°25′ E	7020	1.6	Oligohaline/Mesohaline	Brackish
Dołgie Wielkie	54°42′ N, 17°12′ E	136	1.4	Limnetic	Freshwater
Sarbsko	54°46′ N, 17°38′ E	610	1.2	Limnetic	Freshwater
Ptasi Raj	54°22′ N, 18°48′ E	53	1.2	Mesohaline	Brackish

**Table 2:** Oligonucleotide probes used in the study.

Probe	Sequence	Target rRNA	Specificity	FA **
				[%]
EUB338	5’-GCT GCC TCC CGT AGG AGT-3’	16S	Most bacteria	35
EUB338II	5’-GCA GCC ACC CGT AGG TGT-3’	16S	*Planctomycetales*	35
EUB338III	5’-GCT GCC ACC CGT AGG TGT-3’	16S	*Verrucomicrobiales*	35
NON338	5’-ACT CCT ACG GGA GGC AGC-3’	16S	Control probe complementary to EUB338	35
ALF968	5’-GGT AAG GTT CTG CGC GTT-3’	16S	*Alphaproteobacteria*, except of *Rickettsiales*	20
BET42a	5’-GCC TTC CCA CTT CGT TT-3’	23S	*Betaproteobacteria*	35
	5’-GCC TTC CCA CAT CGT TT-3’ *			
GAM42a	5’-GCC TTC CCA CAT CGT TT-3’	23S	*Gammaproteobacteria*	35
	5’-GCC TTC CCA CTT CGT TT-3’ *			
DELTA495a	5’-AGT TAG CCG GTG CTT CCT-3’	16S	Most *Deltaproteobacteria*	35
	5’-AGT TAG CCG GTG CTT CTT-3’ *		and most *Gemmatimonadetes*	
DELTA495b	5’-AGT TAG CCG GCG CTT CCT-3’	16S	Some *Deltaproteobacteria*	35
	5’-AGT TAG CCG GCG CTT CKT-3’ *			
DELTA495c	5’-AAT TAG CCG GTG CTT CCT-3’	16S	Some *Deltaproteobacteria*	35
	5’-AAT TAG CCG GTG CTT CTT-3’ *			
CF319a	5’-TGG TCC GTG TCT CAG TAC-3’	16S	Most *Flavobacteria*, some *Bacteroidetes*, some *Sphingobacteria*	35
HGC69a	5’-TAT AGT TAC CAC CGC CGT-3’	23S	*Actinobacteria* (high G+C Gram-positive bacteria)	25
	5’-TAT AGT TAC GGC CGC CGT-3’ *			

* Unlabelled competitor ** Formamide FA [%]: formamide concentration in the hybridisation buffer to ensure specific detection of target organisms.

**Table 3:** Mean values (±SD—standard deviation) of physico-chemical parameters of water. Statistically significant differences between groups of lakes are depicted with superscripts (^a^, ^b^, ^c^); (One-way ANOVA, Tukey’s HSD test as a post hoc procedure, *p* < 0.05). Denotations: DO = dissolved oxygen; TOC = total organic carbon; DOC = dissolved organic carbon; EC = conductivity; TIN = total inorganic nitrogen, TP = total phosphorus. N—number of samples analysed; *—median.

		Brackish (N = 90)		Transitional (N = 90)		Freshwater (N = 102)	
		Mean	±SD	Mean	±SD	Mean	±SD
EC	µS/cm	6731 ^c^	3567	1802 ^b^	1150	342 ^a^	254
pH *	-	8.43 ^a^	-	8.80 ^c^	-	8.65 ^b^	-
DO	%	92.05 ^a^	27.37	113.68 ^c^	24.05	102.18 ^b^	22.34
Chl-*a*	µg/L	23.26 ^a^	32.84	30.601 ^a^	46.56	71.31 ^b^	132.97
Salinity	PSU	5.02 ^c^	2.83	1.17 ^b^	0.86	0.24 ^a^	0.15
TOC	mg/L	12.74 ^a^	5.05	20.02 ^b^	13.87	21.623 ^b^	13.97
DOC	mg/L	7.18 ^a^	3.23	11.55 ^b^	6.91	12.11 ^b^	5.29
N-NO_2_^−^	mg/L	0.006 ^b^	0.001	0.006 ^b^	0.001	0.005 ^a^	0.001
N-NO_3_^−^	mg/L	0.89 ^b^	0.73	0.76 ^b^	0.58	0.55 ^a^	0.42
N-NH_4_^+^	mg/L	0.37 ^b^	0.32	0.378 ^b^	0.37	0.19 ^a^	0.13
TIN	mg/L	1.25 ^b^	0.72	1.14 ^b^	0.61	0.75 ^a^	0.40
TP	mg/L	0.40	0.24	0.34	0.28	0.38	0.25
P-PO_4_^3−^	mg/L	0.14 ^b^	0.139	0.10 ^a^	0.080	0.13 ^b^	0.094

**Table 4:** Share of TBN (%) of groups of bacteria and total bacteria number (TBN × 10^6^ cells/mL) in coastal lake types. Significant differences (one-way ANOVA, post hoc: Tukey’s HSD test; *p* < 0.05) are depicted with superscripts (^a^, ^b^, ^c^).

	Freshwater (N = 102)		Transitional (N = 90)		Brackish (N = 90)	
	Mean	±SD	Mean	±SD	Mean	±SD
*Alphaproteobacteria*	4.37 ^c^	1.12	5.27 ^b^	1.94	6.70 ^a^	1.47
*Betaproteobacteria*	17.25 ^a^	4.70	16.13 ^a^	6.33	12.22 ^b^	3.74
*Gammaproteobacteria*	9.51 ^b^	3.80	11.42 ^a^	3.11	12.23 ^a^	3.03
*Deltaproteobacteria*	10.27 ^a^	3.20	12.33 ^b^	5.09	10.10 ^a^	3.74
*Cytophaga-Flavobacterium*	15.21 ^a^	4.67	14.06 ^a^	4.81	14.09 ^a^	4.54
*Actinobacteria*	30.07 ^a^	6.83	31.79 ^a^	5.74	26.82 ^b^	10.05
Total (TBN × 10^6^ cells/mL)	6.93 ^b^	3.58	5.48 ^a^	2.91	4.79 ^a^	2.26

**Table 5:** Similarity percentage analysis (SIMPER) for coastal lake types based on bacteria group contribution in a Bray–Curtis dissimilarity matrix (*Actino*: *Actinobacteria*; *Beta-*: *Betaproteobacteria*; *C-F*: *Cytophaga-Flavobacteria*; *Delta-*: *Deltaproteobacteria*; *Gamma-*: *Gammaproteobacteria*; *Alpha-*: *Alphaproteobacteria*).

		Groups of Bacteria					
		*Actino*	*Beta-*	*C-F*	*Delta-*	*Gamma-*	*Alpha-*
Average dissimilarity		5.0	3.5	3.14	2.85	2.38	1.3
Contribution %		27.6	19.3	17.23	15.65	13.04	7.2
Cumulative %		27.6	46.9	64.2	79.8	92.9	100.0
Mean	Freshwater	30.1	17.3	15.2	10.3	9.51	4.4
	Transitional	31.8	16.1	14.1	12.3	11.4	5.3
	Brackish	26.8	12.2	14.1	10.1	12.2	6.7

**Table 6:** Indicator Species Analysis (ISA) of bacteria groups for habitat types of coastal lakes.

Bacteria Group	Group Identifier for Group with Maximum Observed	Observed Indicator Value (IndVal)	IndVal from Randomised Groups		
			Mean	±SD	*p*
*Alphaproteobacteria*	brackish	41.0	34.3	0.72	0.0002
*Betaproteobacteria*	freshwater	37.8	34.4	0.76	0.0004
*Deltaproteobacteria*	transitional	37.7	34.5	0.80	0.0004
*Gammaproteobacteria*	brackish	36.9	34.3	0.72	0.0002
*Actinobacteria*	transitional	35.8	34.1	0.65	<0.002
*Cytophaga-Flavobacteria*	freshwater	35.1	34.3	0.72	0.08
Mean		37.4	34.3	0.73	0.014

## Data Availability

Not applicable.

## Supplementary Materials

The following supporting information can be downloaded at: https://www.mdpi.com/article/10.3390/ijerph19084592/s1: Table S1: Water quality parameters in relation to lake type and season; Table S2: Similarity percentage analysis (SIMPER) for costal lakes types based on bacteria group contribution in a Bray–Curtis dissimilarity matrix.
