# Supplementary material for: Salinity as a Determinant Structuring Microbial Communities in Coastal Lakes
Source: Int J Environ Res Public Health. 2022 Apr 11;19(8):4592. doi: 10.3390/ijerph19084592 (PMC9028135; doi:10.3390/ijerph19084592)
Supplement: Supplementary file 1 [file ijerph-19-04592-s001.zip › Table S1.pdf]

Water quality parameters in relation to lake type and season

Factor 1	Factor 2	Temp	Temp	Temp	Temp	Temp	pH	pH	pH
**Season**	**Type**	**Mean**	**N**	**Min.**	**Max**	**SD**	**Mean**	**N**	**Min.**
**autumn**	freshwater	13.91	34	11.4	15.2	0.84	8.57	34	7.18
	transitional	14.6	30	12.3	15.97	0.93	8.95	30	8.01
	brackish	13.22	30	6.9	16.6	2.76	8.44	30	7.34
	Total	13.91	94	6.9	16.6	1.79	8.65	94	7.18
**spring**	freshwater	13.99	34	12.1	16.2	0.81	8.65	34	7.91
	transitional	14.83	30	12.77	17.2	1.13	8.63	30	7.98
	brackish	14.24	30	10.5	19	1.98	8.41	30	7.34
	Total	14.34	94	10.5	19	1.41	8.57	94	7.34
**summer**	freshwater	20.9	34	13.1	28.2	5.16	8.74	34	7.86
	transitional	21.05	30	14.6	28.4	4.92	8.83	30	7.99
	brackish	20.39	30	13.7	25.9	4.04	8.45	30	7.26
	Total	20.79	94	13.1	28.4	4.7	8.67	94	7.26

Microbiological parameters in relation to lake type and season

Factor 1	Factor 2	Alpha	Alpha	Alpha	Alpha	Alpha	Beta	Beta	Beta
**Season**	**Type**	**Mean**	**N**	**Min.**	**Max**	**SD**	**Mean**	**N**	**Min.**
**autumn**	freshwater	3.74	34	2	5	0.78	19.2	34	11
	transitional	4.97	30	2.5	9	1.7	15.61	30	6
	brackish	6.45	30	4	9	1.34	11.94	30	6.5
	Total	5	94	2	9	1.71	15.74	94	6
**spring**	freshwater	4.21	34	3	7	0.84	15.5	34	10
	transitional	5	30	3	8	1.85	14.65	30	7
	brackish	6.22	30	4	9	1.23	10.62	30	3.5
	Total	5.1	94	3	9	1.58	13.67	94	3.5
**summer**	freshwater	5.17	34	3	8	1.2	17.06	34	8
	transitional	5.85	30	1	10	2.16	18.13	30	6
	brackish	7.43	30	5	10	1.57	14.08	30	6
	Total	6.11	94	1	10	1.91	16.45	94	6

pH	pH	DO%	DO%	DO%	DO%	DO%	Chl-a	Chl-a	Chl-a	Chl-a
Max	SD	Mean	N	Min.	Max	SD	Mean	N	Min.	Max
9.45	0.4	103.78	34	78.85	145.4	17.17	67.41	34	5.4	670.66
10.09	0.53	119.25	30	88.87	157.6	15.46	32.65	30	5.63	236.56
9.4	0.51	97.1	30	47.6	141.49	23.06	25.04	30	5.56	204.76
10.09	0.52	106.59	94	47.6	157.6	20.7	42.79	94	5.4	670.66
9.93	0.38	97.72	34	59.6	130.5	17.89	62.62	34	5.66	610.13
9.17	0.29	104.36	30	71.3	125	16.21	31.85	30	5.56	234.04
8.8	0.36	97.33	30	11.4	188.14	28.13	20.55	30	5.75	141.85
9.93	0.36	99.72	94	11.4	188.14	21.27	39.38	94	5.56	610.13
9.99	0.36	105.06	34	46.1	160.6	29.67	83.9	34	5.4	569
9.42	0.33	117.43	30	62.3	212.9	33.72	27.32	30	5.51	237.36
9.24	0.5	81.7	30	7.04	126.8	28.46	24.2	30	5.63	157.91
9.99	0.43	101.55	94	7.04	212.9	33.65	46.79	94	5.4	569

Beta	Beta	Gamma	Gamma	Gamma	Gamma	Gamma	Delta	Delta	Delta	Delta
Max	SD	Mean	N	Min.	Max	SD	Mean	N	Min.	Max
30	4.8	7.86	34	2	18	4.62	13.2	34	5	17
36.65	5.91	12.12	30	6.8	19	2.78	13.66	30	6	20
17	2.59	12.35	30	7	18	2.94	11.04	30	5	17.5
36.65	5.49	10.65	94	2	19	4.14	12.66	94	5	20
28	4.43	11.03	34	4	22	3.51	8.11	34	3.95	14
25	4.03	11.24	30	6	22	3.77	10.94	30	1	20
19	3.49	11.56	30	7.5	20.2	3.42	8.22	30	2	18
28	4.51	11.27	94	4	22	3.53	9.05	94	1	20
27	4.3	9.63	34	2	14	2.5	9.51	34	5.45	15
42.5	8.08	10.9	30	6.5	15.65	2.63	12.38	30	2	20
24	4.2	12.76	30	8	19	2.68	11.04	30	3	18
42.5	5.94	11.04	94	2	19	2.88	10.92	94	2	20

Chl-a	EC	EC	EC	EC	EC	Salinity	Salinity	Salinity	Salinity	Salinity
SD	Mean	N	Min.	Max	SD	Mean	N	Min.	Max	SD
131.67	214.62	34	56.7	514.3	163.46	0.21	34	0.02	0.9	0.21
48.03	1200.25	30	105.9	2967.8	1078.16	1.02	30	0.14	2.12	0.57
40.12	5351.42	30	69.3	12929.4	5306.72	4.06	30	0.41	7.41	2.15
87.92	2168.58	94	56.7	12929.4	3757.61	1.7	94	0.02	7.41	2.08
118	137.63	34	59.6	256	61.53	0.1	34	0.02	0.28	0.09
48.75	797.31	30	71.3	1742.33	659.78	0.63	30	0.11	1.32	0.33
26.55	3939.71	30	66.7	12158.8	4561.74	2.92	30	0.3	6.99	2.12
78.94	1561.59	94	59.6	12158.8	3062.7	1.17	94	0.02	6.99	1.71
150.33	194.79	34	46.1	455.8	133.27	0.16	34	0.02	0.9	0.17
44.17	1121.22	30	62.34	2848.3	1057.81	0.98	30	0.11	3.29	0.75
31.37	4908.47	30	54	13228.3	4978.63	3.8	30	1.26	7.65	2.15
98.61	1994.82	94	46.1	13228.3	3500.53	1.58	94	0.02	7.65	2.02

Delta	C-F	C-F	C-F	C-F	C-F	ACTINO	ACTINO	ACTINO	ACTINO	ACTINO
SD	Mean	N	Min.	Max	SD	Mean	N	Min.	Max	SD
2.23	18.97	34	7.25	25	3.3	30.86	34	23	37.5	4.63
4.32	16.36	30	10	26.65	5.05	33.15	30	25	38.65	3.18
3.16	14.7	30	7	24	3.98	28.05	30	10.5	38	7.87
3.46	16.78	94	7	26.65	4.47	30.69	94	10.5	38.65	5.85
3.01	12.94	34	4.65	20.25	3.93	29.07	34	12	36	6.33
5.24	12.47	30	5	22	4.47	30.42	30	22	35	3.98
3.72	12.5	30	7	21	4.71	30.02	30	1	69.98	11.09
4.22	12.65	94	4.65	22	4.32	29.8	94	1	69.98	7.61
2.18	13.71	34	7.4	22	4.28	30.28	34	7	45.8	8.93
5.45	13.36	30	5	21	4.1	31.8	30	14	53	8.44
3.69	15.05	30	9	25	4.62	22.38	30	1	35	9.61
4.07	14.03	94	5	25	4.35	28.24	94	1	53	9.8

TOC	TOC	TOC	TOC	TOC	DOC	DOC	DOC	DOC	DOC	N-NO2
Mean	N	Min.	Max	SD	Mean	N	Min.	Max	SD	Mean
24.86	34	6.78	69.94	14.93	14.08	34	4.16	22.39	5.25	0.0041
22.09	30	5.84	70.01	16.06	11.5	30	2.75	36.58	6.35	0.0062
12.53	30	4.75	22.34	4.17	6.94	30	3.46	12.41	2.13	0.0059
20.04	94	4.75	70.01	13.89	10.98	94	2.75	36.58	5.71	0.0053
17.25	34	4.67	54.12	11.99	9.92	34	3.74	24.24	5.23	0.0066
14.73	30	3.16	32.62	6.93	9.64	30	3	21.11	4.2	0.0069
10.52	30	2.44	19.55	4.41	5.71	30	1.78	12.78	2.62	0.0071
14.3	94	2.44	54.12	8.94	8.49	94	1.78	24.24	4.59	0.0069
22.78	34	6.26	60.07	14.09	12.34	34	4.31	22.42	4.67	0.0049
23.24	30	5.76	86.9	15.5	13.52	30	2.98	42.93	8.99	0.0061
15.17	30	4.72	29.19	5.49	8.89	30	2.96	18.34	3.9	0.0055
20.5	94	4.72	86.9	12.97	11.62	94	2.96	42.93	6.44	0.0055

EUB I-III	EUB I-III	EUB I-III	EUB I-III	EUB I-III	TBNx106	TBNx106	TBNx106	TBNx106	TBNx106
Mean	N	Min.	Max	SD	Mean	N	Min.	Max	SD
83.99	34	55.91	98.96	12.14	7.02	34	1.12	13.5	3.63
81.47	30	54.81	98.91	13	4.09	30	1.1	10.75	2.49
74.72	30	47.96	95	10.09	3.77	30	1.75	8.5	1.92
80.23	94	47.96	98.96	12.34	5.05	94	1.1	13.5	3.16
69.59	34	46.33	99.24	13.31	6.67	34	3.95	13.39	2.01
71.86	30	42	97.71	14.6	4.96	30	0.9	12.56	2.51
70.37	30	57.22	95.78	10.45	5.6	30	1.55	17.56	3.45
70.56	94	42	99.24	12.82	5.78	94	0.9	17.56	2.76
75.34	34	43.31	98.57	13.92	7.11	34	2.45	24.56	4.69
80.91	30	48.63	99.92	15.65	7.4	30	2.95	13.45	2.72
79.77	30	53.86	98	12.92	5	30	2.07	9.5	1.67
78.53	94	43.31	99.92	14.26	6.53	94	2.07	24.56	3.48

N-NO2	N-NO2	N-NO2	N-NO2	N-NO3	N-NO3	N-NO3	N-NO3	N-NO3	N-NH4	N-NH4
**N**	**Min.**	**Max**	**SD**	**Mean**	**N**	**Min.**	**Max**	**SD**	**Mean**	**N**
34	0.0002	0.0162	0.0042	0.63	34	0.1	1.5	0.37	0.18	34
30	0.0039	0.0087	0.0013	0.95	30	0.3	2.55	0.56	0.46	30
30	0.0026	0.0155	0.0028	1.25	30	0.16	3.07	0.78	0.32	30
94	0.0002	0.0162	0.0032	0.93	94	0.1	3.07	0.63	0.31	94
34	0.0007	0.0105	0.0031	0.78	34	0.26	1.83	0.44	0.17	34
30	0.0032	0.0105	0.0029	1.02	30	0.24	2.81	0.61	0.23	30
30	0.0035	0.0155	0.0031	1.1	30	0.42	2.74	0.68	0.21	30
94	0.0007	0.0155	0.003	0.96	94	0.24	2.81	0.59	0.2	94
34	0.0009	0.0121	0.0023	0.25	34	0.03	0.8	0.22	0.23	34
30	0.0034	0.0125	0.0025	0.31	30	0.02	0.78	0.19	0.44	30
30	0.0033	0.0136	0.0023	0.32	30	0.05	1.14	0.24	0.55	30
94	0.0009	0.0136	0.0024	0.29	94	0.02	1.14	0.22	0.4	94

N-NH4	N-NH4	N-NH4	TIN	TIN	TIN	TIN	TIN	P-PO4	P-PO4	P-PO4
**Min.**	**Max**	**SD**	**Mean**	**N**	**Min.**	**Max**	**SD**	**Mean**	**N**	**Min.**
0.01	0.65	0.12	0.81	34	0.16	1.66	0.36	0.13	34	0.02
0.01	1.33	0.43	1.42	30	0.41	2.65	0.58	0.09	30	0.01
0.01	1.02	0.25	1.58	30	0.37	3.29	0.78	0.13	30	0.01
0.01	1.33	0.31	1.25	94	0.16	3.29	0.67	0.12	94	0.01
0.01	0.33	0.08	0.95	34	0.41	1.96	0.41	0.15	34	0.02
0.02	0.88	0.16	1.25	30	0.53	3.06	0.61	0.13	30	0.01
0.01	0.65	0.15	1.32	30	0.55	2.97	0.7	0.16	30	0.02
0.01	0.88	0.13	1.17	94	0.41	3.06	0.59	0.15	94	0.01
0.03	0.58	0.16	0.48	34	0.13	1.08	0.27	0.12	34	0.01
0.01	1.37	0.42	0.76	30	0.15	1.65	0.46	0.08	30	0.02
0	1.33	0.42	0.87	30	0.15	1.79	0.47	0.14	30	0.02
0	1.37	0.37	0.69	94	0.13	1.79	0.43	0.12	94	0.01

P-PO4	P-PO4	TP	TP	TP	TP	TP
**Max**	**SD**	**Mean**	**N**	**Min.**	**Max**	**SD**
0.37	0.09	0.37	34	0.06	1.03	0.21
0.38	0.08	0.28	30	0.05	1.69	0.3
0.48	0.12	0.41	30	0.1	0.89	0.24
0.48	0.1	0.35	94	0.05	1.69	0.26
0.4	0.09	0.42	34	0.17	1.09	0.24
0.3	0.09	0.44	30	0.11	1.16	0.31
0.55	0.15	0.37	30	0.11	1.37	0.23
0.55	0.11	0.41	94	0.11	1.37	0.26
0.37	0.1	0.35	34	0.05	1.06	0.28
0.28	0.07	0.29	30	0.03	0.95	0.2
0.46	0.15	0.41	30	0.11	0.98	0.25
0.46	0.11	0.35	94	0.03	1.06	0.25
