# Supplementary material for: Salinity as a Determinant Structuring Microbial Communities in Coastal Lakes
Source: Int J Environ Res Public Health. 2022 Apr 11;19(8):4592. doi: 10.3390/ijerph19084592 (PMC9028135; doi:10.3390/ijerph19084592)
Supplement: Supplementary file 1 [file ijerph-19-04592-s001.zip › Table S2 Simper .pdf]

Table S2 Similarity percentage analysis (SIMPER) for costal lakes types based on bacteria group contribution in a Bray-Curtis dissimilarity matrix

**Parameter**			**Microbiological group**						**Average dissimilarity**
			**Actino**	**Beta**	**C-F**	**Delta**	**Gamma**	**Alpha**	
**Average** dissimilarity			129.1	62.88	45.27	36.87	25.66	6.94	**306.72**
**Contribution %**			42.09	20.5	14.76	12.02	8.34	2.26	100.00
**Cumulative %**			42.09	62.59	77.35	89.37	97.74	100	469.14
**Mean**	**Transitional**	Gardno	29.4	11.4	12.8	7.05	10.7	7.33	78.68
		Kopań	32.8	17.9	18.1	13.6	12.6	4.33	99.33
		Liwia Łuza	33.2	19.1	11.2	16.3	11	4.14	94.94
	**Brackish**	Łebsko	25.0	14.1	16.6	12.5	11.2	6.97	86.37
		Ptasi Raj	23.6	9.81	11.0	6.92	14.3	5.88	71.51
		Resko	31.6	11.9	13.5	9.81	11.8	7.03	85.64
	**Freshwater**	Sarbsko	28.8	14.3	14	10	8.97	4	80.07
		Wicko	30.9	15.7	14	9.96	10.4	4.38	85.34
		Dołgie Wlk	30.2	22.4	18.1	10.9	8.74	4.73	95.07
